# FdC1 and Leaf-Type Ferredoxins Channel Electrons From Photosystem I to Different Downstream Electron Acceptors

**DOI:** 10.3389/fpls.2018.00410

**Published:** 2018-04-04

**Authors:** Xiaoqian Guan, Shuai Chen, Chia Pao Voon, Kam-Bo Wong, Mikko Tikkanen, Boon L. Lim

**Affiliations:** ^1^School of Biological Sciences, The University of Hong Kong, Pokfulam, Hong Kong; ^2^School of Life Sciences, The Chinese University of Hong Kong, Shatin, Hong Kong; ^3^State Key Laboratory of Agrobiotechnology, The Chinese University of Hong Kong, Shatin, Hong Kong; ^4^Department of Biochemistry and Food Chemistry, Molecular Plant Biology, University of Turku, Turku, Finland

**Keywords:** chloroplast, electron transfer, FdC1, ferredoxin, FTR, photosystem

## Abstract

Plant-type ferredoxins in Arabidopsis transfer electrons from the photosystem I to multiple redox-driven enzymes involved in the assimilation of carbon, nitrogen, and sulfur. Leaf-type ferredoxins also modulate the switch between the linear and cyclic electron routes of the photosystems. Recently, two novel ferredoxin homologs with extra C-termini were identified in the Arabidopsis genome (AtFdC1, AT4G14890; AtFdC2, AT1G32550). FdC1 was considered as an alternative electron acceptor of PSI under extreme ferredoxin-deficient conditions. Here, we showed that FdC1 could interact with some, but not all, electron acceptors of leaf-type Fds, including the ferredoxin-thioredoxin reductase (FTR), sulfite reductase (SiR), and nitrite reductase (NiR). Photoreduction assay on cytochrome *c* and enzyme assays confirmed its capability to receive electrons from PSI and donate electrons to the Fd-dependent SiR and NiR but not to the ferredoxin-NADP^+^ oxidoreductase (FNR). Hence, FdC1 and leaf-type Fds may play differential roles by channeling electrons from photosystem I to different downstream electron acceptors in photosynthetic tissues. In addition, the median redox potential of FdC1 may allow it to receive electrons from FNR in non-photosynthetic plastids.

## Introduction

Located on the stromal side of the thylakoid membrane, plant-type ferredoxins (Fds) belong to a family of low molecular weight proteins with one simple [2Fe–2S] cluster (in the range of -200 to -450 mV) bound to four conserved cysteine residues (Fukuyama, 2004). During photosynthesis, Fds transfer electrons from PSI to a wide range of soluble enzymes, including FNR, FTR, NiR, glutamine-oxoglutarate aminotransferase (GOGAT), SiR, fatty acid desaturase, chlorophyll *a* oxygenase (CAO), *etc.* (Hanke and Mulo, 2013). Hence, Fds play roles in the assimilation of carbon, nitrogen, and sulfur; the synthesis of amino acids, fatty acids, chlorophyll, and phytochromes; and even in the protection of plants from reactive oxygen species (ROS) via the Mehler reaction (Hanke and Mulo, 2013). Multiple isoforms of Fds are found in cyanobacteria, algae, and higher plants (Bertini et al., 2002). They are divided into photosynthetic (leaf) and heterotrophic (root) types based on their localizations, sequences, and functional differences. The leaf-type Fds that are present in chloroplasts receive electrons derived from water during photosynthesis, while the root-type Fds that are present in non-photosynthetic plastids display a more positive redox potential and receive electrons from root-type FNRs, which collect electrons from NADPH derived from the oxidative pentose phosphate (OPPP) pathway to maintain the efficiency of assimilation under a heterotrophic condition (Onda et al., 2000; Hanke et al., 2004a,b). Recently, many novel interacting partners of ferredoxin have been detected by the large-scale screening of potential candidates for electron acceptors, which has expanded our knowledge of the functionalities of ferredoxins (Hanke et al., 2011; Peden et al., 2013).

In the Arabidopsis genome, four nucleus-encoded isoproteins of ferredoxin, namely, AtFd1 (AT1G10960), AtFd2 (AT1G60950), AtFd3 (AT2G27510), and AtFd4 (AT5G10000) were found. These four isoforms represent 7, 90, 3, and 0.05% of the total leaf ferredoxin (Hanke et al., 2004a). AtFd1 and AtFd2 carry specific residues of photosynthetic ferredoxins (Hanke et al., 2004a). AtFd3 encodes one classic root-type sequence (Wang et al., 2000). However, the long evolutionary distance and residue differences made it unclear whether to categorize AtFd4 as a root-type Fd (Hanke et al., 2004a). Although the two leaf-type isoforms share high amino acid identities and interacting capacities with FNR (Hanke et al., 2004b), they were shown to have divergent functions in modulating electron partitioning in the photosystems: AtFd1 (minor form) may preferentially function in the cyclic electron flow while AtFd2 (major form) may preferentially participate in the linear electron flow, although this speculation remains controversial (Yamamoto et al., 2006; Hanke and Hase, 2008; Lehtimäki et al., 2010; Blanco et al., 2013).

In addition to the four well-studied ferredoxin variants, two novel ferredoxin-like homologs, named FdC1 (AT4G14890) and FdC2 (AT1G32550), are encoded in the Arabidopsis genome and share some similarities with the Fds (Hanke et al., 2004a; Goss, 2014). Both proteins carry extra C-termini (9 amino acids for FdC1 and 27 amino acids for FdC2) that are located very near to the redox center (Hanke and Mulo, 2013). First described by Voss et al. (2011), FdC1 in Arabidopsis is a chloroplast-targeted [2Fe–2S] cluster-containing protein that is capable of receiving electrons from the PSI but could not support the photoreduction of NADP^+^ via FNR because of its more positive redox potential. With the increased mRNA level in both the *fd2* knockdown and *fd2* knockout lines, FdC1 was considered as an alternative electron acceptor to PSI under conditions of Fd2 deficiency. However, the downstream electron acceptors of this novel homolog were not identified in this initial study. The physiological functions of both FdCs were then further discussed in a study by Goss (2014). The failure of identifying classical Fd-dependent enzymes in affinity chromatography and yeast two-hybrid experiments have led to the conclusion that both FdCs could not transfer electrons to the classical Fd-dependent enzymes. Based on the identified interacting partners, the authors suggested that FdC1 might play a role in sulfur assimilation and fatty acid synthesis, and FdC2 was likely involved in copper metabolism and maintenance of the chloroplastic grana stacks and chlorophyll, which is similar to its reported homolog in rice (Li et al., 2015; Zhao et al., 2015). Nonetheless, the details regarding the functions of FdCs in the electron transfer in the classical Fd-dependent metabolism remained obscure. Therefore, this study was performed to compare the biological functions of FdC1 with the leaf-type ferredoxins.

## Materials and Methods

### Plant Materials and Growth Conditions

Wild-type (WT) *Arabidopsis thaliana* (L.) Heynh. Ecotype Columbia (Col-0) was used. Vernalized seeds were surface-sterilized with 20% (v/v) bleach for 15 min, and grown in the Murashige and Skoog medium (MS) supplemented with 2% (w/v) sucrose. 10-day-old seedlings were transplanted to soil and grown under long-day (LD) conditions (16 h light with intensity 120–150 μmol photons m^-2^s^-1^ at 22°C and 8 h dark at 18°C) in the growth chambers. *Nicotiana tabacum* was grown on sterilized soil under LD conditions.

### Sequence Alignment and Phylogenetic Analysis

The amino acid sequences of Arabidopsis Fds and FdCs and their homologs from 86 species, including cyanobacterium, green algae, and plantae, were retrieved from the protein databases JGI Phytozome 10.3^1^ and KEGG^2^ by using the Protein Basic Local Alignment Search Tool (blastP) with a p-value less than 1e^-20^. The numbers of corresponding homologs of Fds, FdC1, and FdC2 were calculated and shown as a bar plot along with the phylogenetic trees made by the online tree generator, Interactive Tree Of Life (iTOL)^3^ (Letunic and Bork, 2016), according to the NCBI taxonomy. The multiple alignment of the selected sequences was analyzed and annotated by the software BioEdit version 7.2.5 (Ibis Bioscience, Carlsbad, CA, United States). An evolutionary tree of all the candidate homologs of Fds and FdCs in multiple organisms was built by MEGA 7 using the Neighbor-Joining method (Kumar et al., 2016).

### 3D-Structure Modeling

To simulate the three-dimensional protein structures, the predicted mature amino acid sequences of AtFd2 (53–148a.a.) and AtFdC1 (50–154a.a.) based on the multiple alignment with the mature maize Fd (1GAQ) sequence were analyzed and modeled by the online server Phyre2^4^ (Kelley et al., 2015).

### cDNA Synthesis and Plasmids Construction

The total RNA that was extracted from 20-day-old wild-type (WT) Arabidopsis using TRIzol reagents (Invitrogen, United States) was treated with DNase I (Invitrogen, United States) to remove DNA contamination. A first-strand cDNA library was synthesized by M-MLV reverse transcription (Promega, United States) with oligo dT_15_ primers and was then used as a template for the polymerase chain reaction (PCR). The coding sequences of the candidate photosynthetic and non-photosynthetic apparatus subunits were amplified from the cDNA template using specific primers (**Supplementary Table S3**) by *Pfx* polymerase (Invitrogen, United States) and KAPA HiFi^TM^ HotStart ReadyMix (Kapa Biosystems, United States). The restricted PCR products were ligated into corresponding vectors by the T_4_ DNA ligase (NEB, United States).

### Subcellular Localization of FdC1 in Intact Tobacco Leaves

The full-length coding sequence of FdC1 was amplified and cloned into the XbaI site of the pBA002 vector which expresses an enhanced yellow florescence protein (eYFP) under the control of a *CaMV35S* promoter (Kuang et al., 2009). The plasmid containing FdC1 fused to the C-terminus of eYFP was transfected into tobacco leaves by Agrobacterium syringe-infiltration. After incubation in the darkness at 22°C for 24–48 h, the yellow fluorescence and autofluorescence of the chloroplasts were visualized under a Confocal Laser Scanning Microscope (Carl Zeiss LSM 710 NLO [Carl Zeiss, Germany]).

### Histochemical Analysis of GUS Expression

The putative promoter sequence up to the start codon (ATG) of FdC1 was predicted by the AtcisDB algorithm of the Arabidopsis Gene Regulatory Information Server^5^ (Davuluri et al., 2003). The putative promoter regions with different lengths for *FdC1* (1.5 kb, 0.84 kb) were amplified from the genomic DNA and cloned into the pBA002a*-GUS* vector. The constructs were further transformed into *Agrobacterium tumefaciens* strain GV3101 by freeze-thaw transformation (Chen et al., 1994). The *CaMV35S* promoter was cloned into the vector as a positive control, while a blank vector was used as a negative control. The transgenic T_1_ heterozygotes (5–10 independent lines for each construct) were used to detect the histological expression pattern. The seedlings germinated for 1, 3, 5, 7, and 10 days; the flowers, and green siliques from 4 to 5-week-old plants of each transgenic line (10–15 replicates) were stained overnight at 37°C as previously described (Vitha et al., 1995). The tissues were further washed with 70% (v/v) ethanol to remove the chlorophyll, scanned using a Leica DC 300F digital camera, and analyzed with the software IM50 (Version 1.20 Release 19) (Leica Microsystems AG, Switzerland).

### Yeast Two-Hybrid (Y2H)

A Clontech Matchmaker GAL4-based yeast two-hybrid assay (Takara, Japan) was adopted to examine the interactions between the candidate proteins fused with the active-domain (AD) and the Fds and AtFdC1 fused with the binding domain (BD). Corresponding plasmids containing the BD and AD sequences were mixed together with a filtered mixture solution, containing 50% (v/v) PEG3350, 1 M lithium acetate, and 10 mg/ml single strand carrier DNA, and transformed into the competent cells of gold yeast strain treated with 0.1 M lithium acetate. After mixing with 10% (v/v) DMSO, the cells were incubated at 42°C in a water bath for 20 min and chilled on ice for 3 min. The cultures were then spread on the supplement dropout SD medium DDO and incubated at 28°C for 3–4 days. The colonies showing viability by the activation of related reporter genes of *HIS3, ADE2, AUR1-C*, and *MEL1* were selected and cultured overnight. The cultures were diluted to the equal optical density (OD_600_ = 0.2) and 5 μL aliquots were patched on the agar of double, triple, and quadruple drop-out media, respectively (DDO/ -Leucine/ -Tryptophan, TDO/ -Leucine/ -Histidine/ -Tryptophan, QDO/ -Adenine/ -Histidine/ -Leucine/ -Tryptophan) to compare their interacting strengths.

### Bimolecular Fluorescence Complementation (BiFC)

The full-length coding sequences without the stop codons of the selected Fd-interacting proteins and FdC1 were amplified and cloned into the N-termini of truncated eYFP fused vectors, pSPYNE-*35S* (prey proteins) and pSPYCE-*35S* (bait proteins), respectively (Hu et al., 2006). After transforming into the *Agrobacterium tumefaciens* strain GV3101, the agrobacteria were co-infiltrated into the epidermal cell layers of 30-day-old tobacco leaves via syringe injection (Kerppola, 2006). After incubation in darkness for 48 h, the transfected leaves were examined for fluorescence emission under a LSM 710 NLO Confocal Laser Scanning Microscope (Carl Zeiss, Germany).

### Expression and Purification of Recombinant Fd, FdC1, and OAS-TL C

The coding regions of the mature Arabidopsis *O*-acetylserine (thiol) lyase (OAS-TL C) (112–430a.a.), mature AtFd2 (52–148a.a.), and AtFdC1 (50–154a.a.) were cloned into pGEX-6P-1 vector by fusing the GST tags to their N-termini and the plasmids were transformed into the competent cells of the *Escherichia coli* strain BL21 (DE3) pLysS. The expression of the recombinant proteins was induced by 0.5 mM IPTG at 30°C for 12 h in an LB medium containing 100 μg/mL ampicillin and 0.1 mM FeSO_4_. The fusion proteins were purified by a GSTrap FF column (GE Health, United Kingdom), and the GST tag was cleaved by the PreScission protease. For further purification, gel filtration was run on the HiLoad 16/600 Superdex 75pg packed column (GE Health, United Kingdom) with a buffer, containing 20 mM Tris-HCl (pH 7.5), 150–200 mM NaCl, 0.05% (v/v) Tween 20, and 1 mM DTT. Purified Fd2 and FdC1 were concentrated with Amicon^®^ ultra-4 mL centrifugal filters (MERCK MILLIPORE, United States) and were qualified with an extinction coefficient of 9.68 mM^-1^cm^-1^ at 422 nm (Voss et al., 2011). The concentration of OAS-TL was measured by a Bradford assay (Bradford, 1976). Approximate 200 μL of the purified recombinant Fd2 and FdC1 (40 μM) were scanned with the Multiskan^TM^ GO microplate spectrophotometer (Thermo Fisher Scientific, United States) at wavelengths from 290 to 700 nm.

### Fd-Dependent NiR Enzyme Assays

Crude NiR from rosette leaves of 4–5-week-old WT Arabidopsis (Col-0) was extracted as described (Takahashi et al., 2001). NiR assay was performed by adding 20 μg crude plant extract to a reaction solution, containing 50 mM potassium phosphate buffer (pH 7.5), 1 mM NaNO_2_, and increasing concentrations of recombinant Fd2 and FdC1 (0–6 μM), which had been reduced by 15 mM Na_2_S_2_O_4_ in 0.29 M NaHCO_3_. After 10 min of incubation at 30°C, the nitrite content was measured using the Griess Reagent (Sigma-Aldrich, United States).

### Fd-Dependent SiR Enzyme Assays

The rosette leaves of 4–5-week-old WT Arabidopsis (Col-0) were ground into powder with liquid nitrogen, and the crude SiR was extracted as previously described (Li, 2015). In total, 50 μg crude extract was added to a reaction buffer containing 50 mM HEPES-KOH (pH 7.8), 1 mM Na_2_SO_3_, 5 mM OAS, 1 μg of recombinant OAS-TLC, 10 mM DTT, and increasing concentrations of recombinant Fd2 or FdC1 (0–12 μM), which had been reduced by 15 mM Na_2_S_2_O_4_ in 0.29 M NaHCO_3_. After the incubation at 25°C for 20 min, the reaction was stopped by TCA, and the amount of cysteine was qualified as described (Gaitonde, 1967).

### Electron Transfer Assays on NADP^+^ and Cytochrome *c*

To measure the electron transfer ability from PSI to the recombinant Fd2 and FdC1, an assay based on photoreduced cytochrome *c* was carried out in a 96-well plate (Voss et al., 2011). A mixture with a total volume of 200 μL, containing 10 μg/mL thylakoid membrane, 25 mM HEPES-KOH (pH 7.6), 1 mM MgCl_2_, 100 mM NaCl, 200 μM cytochrome *c*, and various concentrations of recombinant Fd2 or FdC1 (0–4 μM), was illuminated under red LED light with an intensity of 300 μmol photons m^-2^ s^-1^. The absorbance for the reduced cytochrome *c* at 550 nm was recorded with the coefficient of 18.5 mM^-1^ cm^-1^. For the assay of the photoreduced NADP^+^, 200 μM NADP^+^ was used instead of cytochrome *c*. The absorbance at 340 nm was monitored for NADPH with a coefficient of 6.22 mM^-1^ cm^-1^. The dark control assays were performed and used for calibration.

### Construction of the Plasmid for FdC1 Overexpression

Since FdC1 T-DNA insertion line is not available from TAIR and FdC1 RNAi lines exhibited wild-type like phenotype (Goss, 2014), we generated the overexpression lines for FdC1 characterization. An overexpression (OE) plasmid pCXSN-FdC1 was constructed, in which the expression of the full-length coding sequence of FdC1 was driven by a *CaMV35S* promoter (Chen et al., 2009). The construct was transformed into the *Agrobacterium tumefaciens* strain GV3101 and then into WT Arabidopsis using the floral dip method (Wiktorek-Smagur et al., 2009). The overexpression lines were selected on MS plates containing 30 μg/mL hygromycin. Their transgenic status was confirmed by western blotting with polyclonal FdC1-specific antibody raised by GenScript (China) and semi-quantitative reverse-transcript PCR (semi RT-PCR). The homozygous T_3_ seeds were used for further experiments.

### Chlorophyll Fluorescence Measurements of the FdC1-Overexpression Lines

Six replicates of 3-week-old plants from three genotypes (WT and OE lines of FdC1) were scanned with IMAGING-PAM M-Series Chlorophyll Fluorometer (Heinz Walz, Germany). All the lines were dark-adapted for 1 h before the determination of F_o_ and F_m_. The chlorophyll fluorescence was monitored for 27 min. The light intensities were increased stepwise every 3 min as follows: 0, 81, 146, 186, 281, 336, 461, 701, and 926 μmol photons m^-2^s^-1^ (PAR). The saturation pulses were applied immediately prior to the increasing light intensities. The average values and standard errors of the photosynthetic electron transport rate (ETR), photochemical quenching (qP), non-photochemical quenching (NPQ), and efficiency of photosystem II (ΦII) against light intensity were analyzed by software ImagingWin (Heinz Walz, Germany) and calculated as previously described (Maxwell and Johnson, 2000). The oxidized P700 was measured with Dual-PAM-100 (Heinz Walz, Germany) as described (Tikkanen et al., 2015).

## Results

### FdC1 Appears Later in Evolution

**FIGURE 1:**
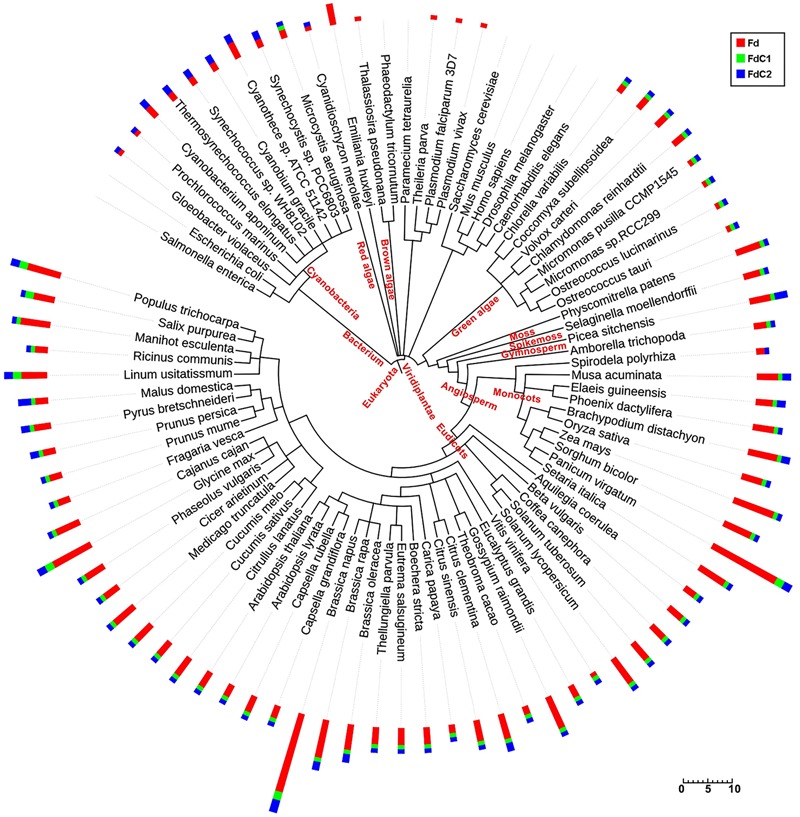
Distribution of Fds and FdCs and their homologs in 87 species. Amino acid sequences retrieved by blastP (*p*-value < e^-20^) from the KEGG and Phytozome databases were used to build the phylogenetic tree. The numbers of the corresponding homologs of Fd (red), FdC1 (green), and FdC2 (blue) in each species are displayed in the bar plots with scale bar. The figure was drawn online using NCBI taxonomy and Interactive Tree of Life (iTOL).

As shown in the phylogenetic tree (**Figure 1**), plant-type ferredoxins were widely distributed in organisms from prokaryotes (cyanobacteria) and algae (brown, red, and green) to plants (mosses, spike mosses, gymnosperms, and angiosperms). The plant-type Fd-coding genes were found predominantly among the oxygen-evolving photosynthetic organisms, particularly highly vascularized plants, and can be divided into the following four types: higher plant leaf-type Fd in chloroplasts, higher plant root-type in heterotrophic plastids, algal-higher plant conserved Fd with a short C-terminal extension, and cyanobacterial-higher plant conserved Fd with a long C-terminal extension (**Supplementary Figure S1A**). The divergent clusters of the two Fds with the C-terminal extension (FdCs) indicated their discrepant evolutionary features. Interestingly, with differential evolutionary distances, FdC1 started to appear in green algae, while FdC2 could be found in cyanobacteria. The maintenance of FdC2 during evolution implies its essential role in photosynthesis. In addition to the conserved cysteine residues (Hanke et al., 2004a), the C-termini of FdC1 also exhibit high similarities, particularly in higher plants (**Supplementary Figure S1B**).

### FdC1 Is a Widely Expressed Plastid-Targeted Protein

**FIGURE 2:**
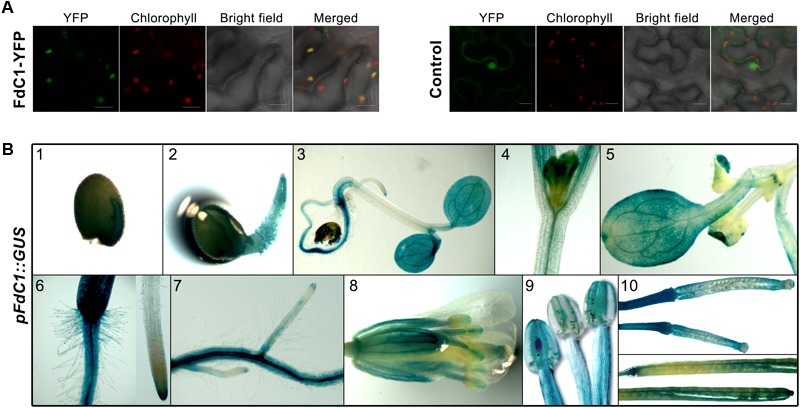
The subcellular localization and expression pattern of FdC1. **(A)** The full-length sequence of FdC1 was fused with YFP and transiently expressed in tobacco leaves. The YFP signal in green was excited at 515 nm. Chlorophyll autofluorescence in red was excited at 458 nm. Merged images of the YFP signal, chlorophyll signal, and bright field are presented in the last panel with a 20 μm scale bar. FdC1 is a chloroplast-targeted protein. **(B)** Histochemical localization of GUS activity in the transgenic Arabidopsis lines under the control of the *FdC1* promoter (1.5 kb) in young seedlings, roots, mature flowers, and siliques. (1) One-day-old seed with GUS staining in both the cotyledon and episperm. (2) Two-day-old seedling with moderate staining in the radicle. (3) Five-day-old seedling with intense GUS staining in the cotyledon leaves, hypocotyl, and roots. The magnified images of the partial 3-day-old hypocotyl (4), and cotyledon (5) and roots (6 and 7) of 10-day-old seedling with intense, diffuse GUS staining. (8) Flower with strong GUS activity in the stigma, sepal, and both long and short stamens. (9) A magnified image of the stamens with GUS staining. (10) Immature siliques (upper) and fore and aft ends of siliques (lower) from mature plants with intense GUS staining in the valves, septa, and pedicels. The GUS activity under the control of 0.84 kb *FdC1* promoter showed an identical expression pattern.

The localization of FdC1 in the chloroplasts was indicated by the overlay of eYFP (green) and autofluorescence of chlorophyll (red) against the irregularly diffused signals of the negative control (**Figure 2A**). Both putative promoter regions of FdC1 drove identical expression patterns *in planta* (**Figure 2B**). Intensive GUS activities in cotyledons, radicles, cotyledon leaves, and hypocotyls were observed at the early stages of seedling establishment. Surprisingly, the strong GUS activity shown in the young roots of the *FdC1-promoter* transgenic lines contradicted its previous description as a leaf-type protein (Voss et al., 2011), and was consistent with its relatively high signals in both the shoot and root microarray data (**Supplementary Table S2**) (Sun et al., 2013). There was a strong expression in both the primary root and lateral roots, together with the root caps of the root tips, but there was only a slight expression in the root hairs. In the reproductive tissues, the GUS staining had an extensive expression in the valves, septum, and pedicel of the young and mature siliques, as well as in the stigma, sepal, and both long and short stamens of the flowers, especially with strong signals in both the anthers and filaments. The transgenic lines that contained the blank pBA002a-*GUS* vector did not exhibit any GUS staining while the GUS driven by *CaMV35S* promoter showed a diffused and wide expression (data not shown).

### FdC1 Shares Most, but Not All, Interacting Partners of the Leaf-Type Fds

**FIGURE 3:**
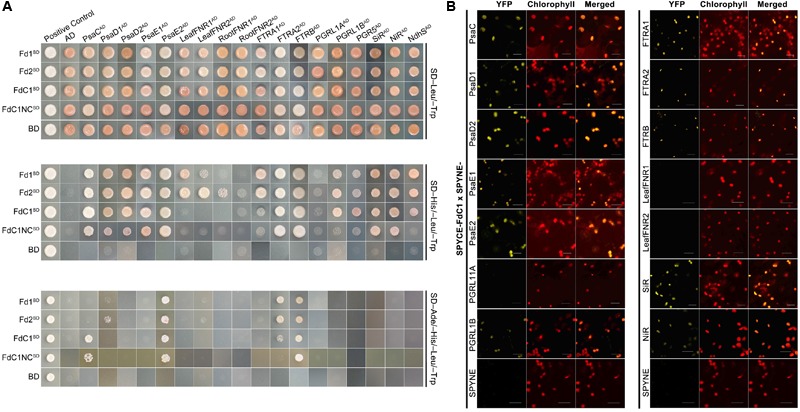
The comparison of the interacting partners between the two leaf-type ferredoxins and FdC1. **(A)** The yeast two-hybrid results showed that FdC1 was able to interact with several, but not all, well-defined interacting partners of leaf-type Fds. FdC1 can interact with PsaC, PsaD1, PsaD2, PsaE1, PsaE2, FTRA2, FTRB, SiR, NiR, NdhS, PGR5, and PGRL1B. Truncation of the C-terminus of FdC1 (1–145a.a.) (labeled as FdC1NC) abolished its interactions with NdhS, PGR5 and PGRL1B. Blank vectors of BD (pGBKT7) and AD (pGADT7) were, respectively, co-transformed with baits and preys to test for auto-activation. The assays were performed in dropout media of DDO (SD/-Leu/-Trp), TDO (SD/-Leu/-Trp/-His), and QDO (SD/-Ade/-His/-Leu/-Trp). The positive control was the co-transformation of plasmids pGBKT7-53 and pGADT7-T while the negative control was the co-transformation of plasmids pGBKT7-Lam and pGADT7-T. **(B)** Verification of the interactions between FdC1 and its binding partners by BiFC after a transient expression in tobacco leaves. Empty pSPYNE-*35S* and pSPYCE-*35S* vectors were used as controls. Reconstituted YFP fluorescence was excited at 515 nm. Chlorophyll autofluorescence was excited at 458 nm. Overlay of the YFP signal and the chlorophyll autofluorescence is shown in the right panel. All images were captured under the same gain settings of corresponding PMT channels and repeated three times (Scale bars: 20 μm). A negative control assay is shown as **Supplementary Figure S2**.

A yeast two-hybrid assay was performed to detect the interactions between FdC1 and the classical docking partners of Fds in multiple Fd-dependent photosynthesis and assimilation pathways. The results (**Figure 3A**) showed that FdC1 interacted with some, but not all, docking partners of the two leaf-type Fds, including the subunits of the upstream electron donor PSI (subunit C, D1, D2, E1, and E2 of photosystem I) and several downstream electron acceptors of Fds (FTR A and catalytic chain B, PGR5, PGR5-like protein B, NADH dehydrogenase-like complex S, SiR, and NiR) but could not interact with the leaf-type and root-type FNRs. The absence of the C-termini (labeled as FdC1NC) did not affect binding properties of FdC1 dramatically. Most of the positive interactions between FdC1 and the candidate proteins at the chloroplasts were confirmed by bimolecular fluorescence complementation (BiFC) (**Figure 3B** and **Supplementary Figure S2**).

### Results of the *in Vitro* Enzymatic Assays

**FIGURE 4:**
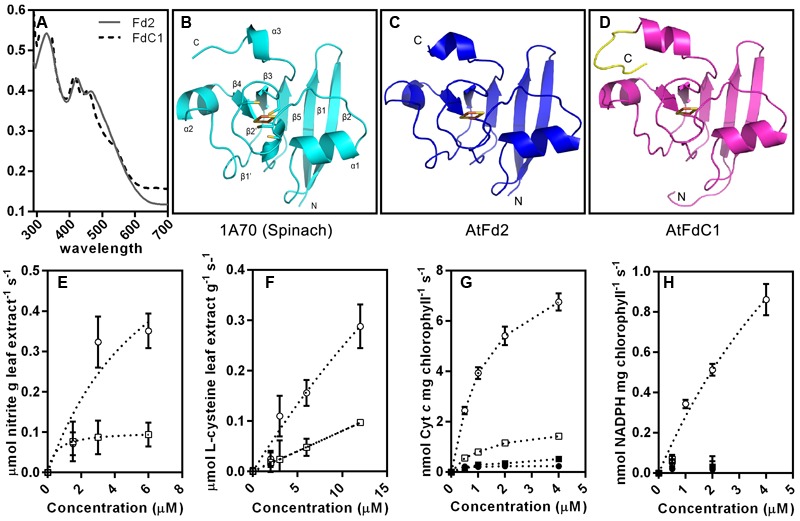
The putative 3D structures of Fd2 and FdC1 and their electron transferring capabilities. **(A)** The UV-Visible absorption spectrum of the recombinant Fd2 and FdC1. 40 μM recombinant Fd2 (gray plain line) and FdC1 (black dotted line) were scanned from 290 to 700 nm. The cartoons are successively the crystal structure of spinach ferredoxin (1A70) (light blue) **(B)**, the computer modeled 3D structures of AtFd2 (blue) **(C)** and AtFdC1 (rose red) **(D)** with its extended C-terminus in yellow. The orientations of the proteins are labeled from N to C. The conserved iron-sulfur clusters ligated by the four cysteines are shown as sticks and red-orange spheres. **(E)** The ability of recombinant Fd2 and FdC1 to transfer electrons to NiR. The consumption of nitrite by the Fd-dependent NiR in the leaf extract was enhanced by adding increasing concentrations (0∼6 μM) of recombinant AtFd2 (open circle) and AtFdC1 (open square). The variance tendency of nitrite is shown as dashed lines. **(F)** The ability of recombinant Fd2 and FdC1 to transfer electrons to SiR. L-cysteine generated by the Fd-dependent SiR with *O*-acetylserine thiol lyase included in the leaf extract was enhanced by adding increasing concentrations (0∼12 μM) of recombinant Fd2 (open circle) and FdC1 (open square). The variance tendency of L-cysteine is shown as dashed lines. **(G)** Assay of electron transfer from PSI (cytochrome *c*). The variance trends of the light-reduction of cytochrome *c* as indicated by the absorbance at 550 nm in 1 min are shown to compare the catalytic abilities of increasing concentrations (0∼4 μM) of Fd2 (open circle) and FdC1 (open square) against the dark control (closed circle for Fd2 and closed square for FdC1). The variance tendency of cytochrome *c* is shown as dashed lines. **(H)** The electron transfer assays on the photoreduction of NADP^+^ with electrons from Fd2 and FdC1 were performed under LED light condition. However, only recombinant Fd2 (open circle) was able to support the reduction of NADP^+^ when exposed to light, and no detectable increase of NADPH was observed when the recombinant FdC1 was used instead. The variance tendency of NADPH is shown as dashed lines. All the results shown here are shown as mean ± standard error (*n* = 3). The positive controls for NiR, SiR, and cytochrome *c* assays performed with gradient concentrations of methyl viologen showed quick response and equilibration (data not shown).

All plant-type ferredoxins have similar overall folding based on homology modeling (Bertini et al., 2002) (**Figure 4**). However, the extra C-terminal extension of FdC1 might affect its conformation (**Figures 4B–D**). Both recombinant Fd2 and FdC1 expressed in *E. coli* showed specific peaks around 420 and 460 nm, a characteristic of [2Fe–2S] clusters (Voss et al., 2011) (**Figure 4A**). The photoreduction of cytochrome *c* and NADP^+^ was performed to detect the photosynthetic capability of FdC1. FdC1 could support the photoreduction of cytochrome *c* but not the photoreduction of NADP^+^ with endogenous FNR at the thylakoid membrane (**Figures 4G,H**). The assay performed in dark confirmed the direct electron donations from Fd2/FdC1 to cytochrome *c.* In the NiR [EC 1.7.7.1] assay, the consumption of nitrite increased as the concentrations of recombinant Fd2 and FdC1 increased but FdC1 exhibited a lower catalytic efficiency than Fd2 (**Figure 4E**). Similarly, in the SiR [EC 1.8.7.1] assay, FdC1 could support SiR activity but displayed a lower efficiency than Fd2 (**Figure 4F**).

### Increased FdC1 Content Affects the Photosynthetic Electron Transfer

**FIGURE 5:**
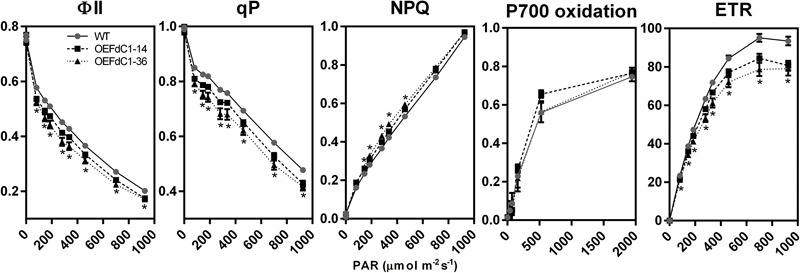
Chlorophyll fluorescence parameters of the wild-type and FdC1 overexpression lines. The comparison between the wild-type (gray closed circle with black plain line) and FdC1 overexpression lines (OEFdC1-14 as black closed square with black dashed line, OEFdC1-36 as black closed triangle with black dotted line) is shown as mean ± standard error. The *t*-test results with significant changes (*p*-value ≤ 0.01) in both overexpression lines are labeled by ^∗^. The measurement of ΦII, qP, NPQ, and ETR had six biological replicates while the measurement of P700 oxidation had three biological replicates.

Overexpression of FdC1 did not cause observable changes in the growth phenotypes (**Supplementary Figure S3**) but significantly affected the chlorophyll fluorescence parameters (**Figure 5**). The photosystem II yield, photochemical quenching, and photosynthetic electron transfer rate were all decreased at light intensities from 81 PAR to 926 PAR, implying a rapid reduction in oxidized PQ and a consequent decrease in the electron transfer capacity of PSII (Genty et al., 1989). The changes were reproducible in two independent OE lines of FdC1. Therefore, FdC1 could be involved in the photosynthetic electron transfer and the flexible regulation of photosynthesis in response to rapid changes in light intensity. However, unlike the enhanced quantum yield of PSI and the electron flux in PSI in the AtFd2 (OE) transplastomic tobacco (Yamamoto et al., 2006), the overexpression of FdC1 showed no significant P700 redox changes from low to high light conditions. Similarly, wild-type like pattern of P700 oxidation was reported in the tobacco overexpressing the minor Pea Fd isoform (Blanco et al., 2013).

## Discussion

The identification of Fd homologs with extended C-termini have expanded the scope of the plant-type ferredoxin family in Arabidopsis. The distribution of total plant-type ferredoxins and the two FdC isoproteins among the numerous photoautotrophic organisms depicted here has supported the functional differentiation of the ferredoxins at an early stage (Fukuyama, 2004). The specific but highly conserved extensions on their C-termini have differentiated both novel homologs during evolution as follows: FdC2 existed in cyanobacteria, while FdC1 appeared later in green algae. The divergent evolutionary distances have expanded the classification of the plant-type ferredoxins to leaf-type, root-type, and Fd-like proteins with long or short extensions on their C-termini. Fds can mediate electron transfer in opposite directions in source and sink tissues. The direction is dependent on the sources of the electrons and the mid-point potentials of Fds and the electron acceptors/donors. During evolution, plant-type Fds have evolved into leaf-type Fds and root-type Fds to mediate electron transfer in each direction (**Figure 1**). In Arabidopsis, the leaf-type and root-type Fds have divergent mid-point potentials (AtFd1, -425 mV; AtFd2, -433 mV; AtFd3, -337 mV; AtFd4, -152 mV) (Hanke et al., 2004a). This allows the leaf-type Fds to receive electrons from PSI in chloroplasts, and the root-type Fds to receive electrons from FNRs in heterotrophic plastids. By contrast, FdC1, which is expressed in both leaves and roots (**Figure 2**), has a median mid-point potential (-281 ± 3 mV) (Voss et al., 2011), which might allow it to mediate electron transfer in different directions in both tissues.

In leaves, PSI mediates the light-driven electron transfer from the lumen-side plastocyanin (PC) to the stromal-side Fd (Fromme et al., 2001). Subsequently, ferredoxin oxidation and NADP^+^ reduction to NADPH will be driven by FNR to support carbon fixation in the Calvin cycle (Andersen et al., 1992). There are 15 core subunits (PSI-A to -L and PSI-N to -P) in PSI (Scheller et al., 2004). Among these subunits, the marginal subunits (C, D, and E) shaped the interrelated structure of the “stromal ridge” of PSI (Nelson and Yocum, 2006). Both PsaD and PsaE conferred docking sites for soluble ferredoxin to PsaC and helped stabilize their interaction (Fromme et al., 2001). The interactions between both leaf-type Fds and FdC1 with all of the components of the “stromal ridge” of PSI (**Figure 3**), particularly PsaD and PsaE, support their ability to receive electrons from PSI (Voss et al., 2011). The varied binding affinities of the alleles of PsaD and PsaE implied their divergent roles although they were assumed to be functionally redundant and identical (Ihnatowicz et al., 2004; Ihnatowicz et al., 2007). The electron transfer assay on the photoreduction of cytochrome *c* at PSI also confirmed the role of FdC1 as an electron acceptor of PSI (**Figure 4G**). In addition to the more positive redox potential of FdC1 than leaf-type Fds (Voss et al., 2011), the failure to form ionic complexes between the FdC1 and leaf-type FNRs would prevent electron transfer between them. The negative results of Y2H and BiFC on FdC1 with the leaf-type FNRs (**Figure 3A**) were consistent with the result of the electron transfer assays on NADP^+^ using thylakoid membrane (**Figure 4H**). The loss of the conserved residues responsible for the Fd-FNR complex conformation in FdC1 (Kurisu et al., 2001; Hanke et al., 2004b), such as D66, D65, and A98, could explain their inability in supporting the photoreduction of NADP^+^ at leaf-type FNRs (**Supplementary Table S1**).

Electrostatic interactions are important for the formation of the 1:1 Fd-NiR complex in spinach (proposed model 2AKJ-1A70) and 1:1 Fd-SiR complex (5H92) in maize (Hirasawa et al., 2009; Kim et al., 2016). The positively charged residues K49, K80, K83, K100, K268, N304, and R502 of NiR and the negatively charged residues E93 and E94 of Fd are involved in the formation of salt bridge network for the binding of NiR and Fd (Hirasawa et al., 2009). These two glutamate residues are conserved in both AtFd2 and FdC1. The basic residues distributed at the interface of the [4Fe–4S] cluster of SiR form intermolecular interactions with the acidic residues of Fd, such as R111 and R114 to D60, R324 to D34, K582 to E29, and K584 to E30 (Kim et al., 2016). All these residues are retained in Arabidopsis Fd2 while FdC1 only contains the conserved residue of D34 (**Supplementary Table S1**). Enzyme assays confirmed the capacity of FdC1 to pass electrons to these two enzymes to support the reduction of sulfite and nitrite (**Figures 4E,F**). The lower catalytic efficiency of FdC1 on passing electrons to SiR and NiR could be explained by its more positive redox potential and the loss of highly conserved residues required for the interactions.

According to the crystal structure of the Fd-FTR binary complex of *Synechocystis* sp. (2PVG), The [2Fe–2S] cluster and the [4Fe–4S] cluster, coordinated by S38 of Fd and C74 and N73 of FTRB, are proposed to be the electron path between Fd and FTRB (Dai et al., 2007). Both AtFd2 and AtFTRB share highly conserved residues that are responsible for the intermolecular interactions with Fd and FTRB of 2PVG, respectively (**Supplementary Table S1**). In addition to the conserved cysteines in the iron-sulfur centers, E92 of Fd, which can form a salt bridge with K47 of FTRB, is conserved in AtFdC1. FdC1 might function as a reducing equivalent donor for the ferredoxin-thioredoxin system to regulate the activities of key, light-activated enzymes in the Calvin-cycle, such as fructose-1,6-bisphosphatase (FBPase), and NADP-dependent malate dehydrogenase (NADP-MDH) (Dai et al., 2000). Although FTRA is not directly involved in the interactions between ferredoxin and FTRB (Dai et al., 2007), the differential binding properties of FTRA1 and FTRA2 implied a functional division of these two alleles. Moreover, the interactions between FdC1 and the core components of the two cyclic electron routes indicated its potential to transfer electrons from PSI to the PGRL1 homodimers/PGRL1-PGR5 heterodimers (Munekage et al., 2002; DalCorso et al., 2008) and NAD (P) H-dehydrogenase (NDH) complex via the NdhS (Yamamoto and Shikanai, 2013). Truncation of the C-terminus of FdC1 abolished its interactions with NdhS, PGR5 and PGRL1B (**Figure 3A**).

**FIGURE 6:**
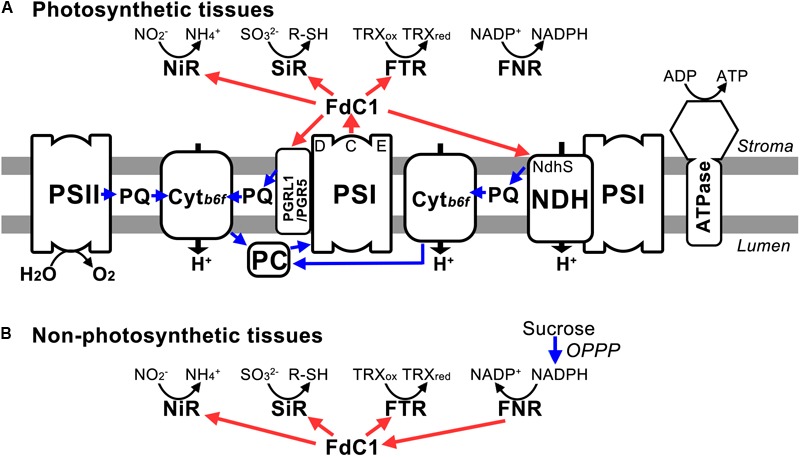
The model of electron transferring pathways mediated by FdC1. **(A)** In photosynthetic tissues, FdC1 accepts electrons from PSI and transfers the electrons to downstream electron acceptors (PGR5, PGRL1B, NdhS, SiR, NiR, FTRA2, and FTRB) other than FNR. **(B)** In non-photosynthetic tissues, it could transfer the electrons from FNR via the OPPP pathway to NiR, SiR, and FTR. Both blue and red arrows indicate electron transfer. The electron transfer pathways potentially facilitated by FdC1 are shown by red arrows.

The overexpression of FdC1 in Arabidopsis did not lead to observable morphological changes. However, the chlorophyll fluorescence parameters of the transgenic lines varied significantly from the WT under various light intensities. The overexpression of FdC1 resulted in a lower electron transfer rate (ETR), PSII yield (ΦII), and photochemical quenching (qP) but higher non-photochemical quenching (NPQ), which indicated that the photochemical system of FdC1 transgenic lines was more sensitive to excitation pressure. Since FdC1 accepted the electrons from PSI, it could competitively divert the electrons from Fd to their downstream electron acceptors, such as from FNRs to NiR, SiR, and FTR, or from the linear electron flow to the cyclic electron flow (**Figure 6A**). The leaf-type FNR has a turnover number of 200–500 s^-1^ (Ceccarelli et al., 2004), which is much higher than the turnover rate of NiR (10–20 s^-1^) (Hirasawa et al., 1993). Overexpression of FdC1 may therefore reduce ETR by reducing the electrons passing to FNR (**Figure 5**).

In roots, root-type Fds transfer electrons from the OPPP pathway to NiR and SiR for nitrogen and sulfur assimilation. Mid-point potentials of the sirohemes of NiR (-270 mV) and SiR (-285 mV) are very close to that of FdC1 (-281 mV) (Hirasawa et al., 2004) and enzymatic assay showed that FdC1 can receive electrons from root-type FNR (Voss et al., 2011). Hence, FdC1 may play an analogous role to root-type Fd in transferring electrons from the OPPP pathway to NiR and SiR in non-photosynthetic tissues (**Figure 6B**). Kinetic assays showed that the Kms of leaf-type FNR with Fd and FdC1 were 2.8 and 27.4 mM, respectively and that of root-type FNR with Fd and FdC1 were 13.8 and 39.9 mM, respectively (Voss et al., 2011). The stronger affinity between Fd and leaf FNR corroborated with our Y2H data (**Figure 3A**). Although interaction between root-type FNR and FdC1 could not be detected by Y2H, enzyme assay showed that FdC1 could receive electrons from root-type FNR (Voss et al., 2011).

In summary, while ferredoxins and their docking enzymes co-evolved from cyanobacteria to higher plants (Sakakibara et al., 2012), FdC1 appeared later in green algae (**Figure 1**) (Goss, 2014). The genome of the smallest green alga *Ostreococcus tauri* only contains a single copy of Fd and FdC1 (**Figure 1**). Our data postulate that Fd and FdC1 divide their labor in diverting electrons to different acceptors in chloroplasts. While Fd rapidly channels electrons to FNR for vigorous carbon fixation, FdC1 can divert electrons to the other electrons-demanding activities, such as nitrite and sulfite reduction. When multicellular photosynthetic organisms evolved to develop both autotrophic and heterotrophic tissues, Fd and FNR were also duplicated and diverged to leaf and root isoforms with differential mid-point potentials, so as to pass electrons in reverse directions in autotrophic and heterotrophic tissues (Pierella Karlusich and Carrillo, 2017). By contrast, in most plant genomes, there is only one single FdC1 gene. Its moderate mid-point potential, which is very close to that of SiR and NiR (Hirasawa et al., 2004), may allow it to transfer electrons from different sources of electrons to SiR and NiR in autotrophic and heterotrophic tissues. Hence, the co-existence of both FdC1 and Fds may allow a more flexible regulation of electron partition to carbon, nitrogen, and sulfur assimilation pathways.

## Accession Numbers

Sequence data from this work can be found in the Arabidopsis Genome Initiative or GenBank/EMBL databases under the following accession numbers: Fd1, AT1G10960; Fd2, AT1G60950; Fd3, AT2G27510; Fd4, AT5G10000; FdC1, AT4G14890; FdC2, AT1G32550; FTRA1, AT5G23440; FTRA2, AT5G08410; FTRB, AT2G04700; LeafFNR1, AT5G66190; LeafFNR2, AT1G20020; NdhS, AT4G23890; NiR, AT2G15620; OAS-TLC, AT3G59760; PGR5-Like1A, AT4G22890; PGR5-Like1B, AT4G11960; PsaC, ATCG01060; PsaD1, AT4G02770; PsaD2, AT1G03130; PsaE1, AT4G02770; PsaE2, AT2G20260; RootFNR1, AT3G05390; RootFNR2, AT1G30510; SiR, AT5G04590.

## Author Contributions

BL designed the study. SC and KW performed the *in silico* modeling of the putative 3D structures. CV designed and helped analyze the measurements of the chlorophyll fluorescence parameters. MT carried out the P700 assay. XG carried out all other experiments and wrote the manuscript.

## Conflict of Interest Statement

The authors declare that the research was conducted in the absence of any commercial or financial relationships that could be construed as a potential conflict of interest.

## Footnotes

1 http://phytozome.jgi.doe.gov/pz/portal.html

2 http://www.genome.jp/kegg/

3 http://itol2.embl.de/index.shtml

4 http://www.sbg.bio.ic.ac.uk/phyre2/html/page.cgi?id=index

5 http://arabidopsis.med.ohio-state.edu/

## Supplementary Material

FIGURE S1 **(A)** Phylogenetic analysis of FdCs. The evolutionary history was inferred using the Neighbor-Joining method of MEGA7. The tree was drawn to scale, with branch lengths in the same units as those of the evolutionary distances. The evolutionary distances were computed using the Poisson correction method and are in the units of the number of amino acid substitution per site. 202 amino acid sequences of the putative mature proteins of the four isoforms of AtFd (Fd1∼Fd4) and two isoforms of AtFdC (FdC1 and FdC2) from 33 species were analyzed. All positions containing gaps and missing data were eliminated. There were in total 86 sequences in the final dataset. The ferredoxin and ferredoxin-like homologs could be divided into four clusters: leaf-type Fd (green dashed line), root-type Fd (dark red dashed line), FdC1-type (orange dashed line), and FdC2-type (blue dashed line). The Fd and FdC isoforms in *A. thaliana* are marked with red dots. **(B)** the multiple alignment of FdC1 from *A. thaliana* and its homologs from the other 28 species. *A. trichopoda*, AmTr_v1.0_scaffold00004.27; *A. thaliana*, AT4G14890.1; *A. coerulea*, Aquca_049_00211.1; *B. stricta*, Bostr.0597s0118.1; *B. rapa*, Brara.A02028.1; *C. rubella*, Carubv10007616m; *C. reinhardtii*, Cre07.g334800.t1.2; *C. arietinum*, Ca_11871; *C. sinensis*, cit_102630095; *C. sativus*, Cucsa.254290.1, *E. salsugineum*, Thhalv10026483m; *F. vesca*, mrna09776.1; *G. max*, Glyma.01G238000.1, Glyma.11G005700.1, *L. usitatissmum*, Lus10006116, Lus10010557; *M. domestica*, MDP0000155343; *M. esculenta*, cassava4.1_018587m; *M. truncatula*, Medtr5g006320.1; *M. pusilla*, 27711; *M. guttatus*, Migut.N00186.1; *O. sativa*, LOC_Os03g45710.1; *O. tauri*, Ot06g03460; *P. trichocarpa*, Potri.010G087300.1, Potri.008G153200.1; *P. persica*, ppa012963m; *S. italica*, Si037632m; *S. tuberosum*, PGSC0003DMT400086140; *T. parvula*, Tp7g13030; *V. vinifera*, GSVIVT01017981001; *V. carteri*, Vocar20007148m; *Z. mays*, GRMZM2G359127_T01. Identical residues are colored in white and highlighted in black background, and the homologous residues are shown in gray background. The conserved residues are showed in red on the top and the highly conserved C-termini are boxed with a dotted red line.

FIGURE S2 Negative control of BiFC. The assay was carried out by co-transforming agrobacteria with empty pSPYCE-*35S* and pSPYNE-*35S* vectors containing the coding sequences of the candidate proteins into tobacco leaves. The same laser scanning settings of **Figure 3B** were used to monitor the reconstituted YFP signals and chlorophyll autofluorescence. No YFP signal was detected (Scale bars: 20 μm).

FIGURE S3 Growth phenotypes of FdC1 overexpression lines. **(A)** The phenotypes of the overexpression (OE) lines of FdC1 (OEFdC1-14 and OEFdC1-36) compared with the wild-type (WT). The vertical views of representative 4-week-old Arabidopsis and side views of representative 7-week-old plants are shown. All the plants were grown under long-day light regimes. **(B)** The semi-quantitative RT-PCR results showing the overexpression of FdC1 in transgenic lines at transcripts level. The primers were listed in **Supplementary Table S3**. The house-keeping gene ubiquitin 11 was chosen as the control. **(C)** The western blotting results showing the overexpression of FdC1 in transgenic lines compared with the wild-type. The Coomassie blue stain of Rubisco in SDS-PAGE gels was used as the control for equal loading.

TABLE S1 Interacting amino acid residues of the two interacting proteins in resolved crystal structures and their corresponding residues in Arabidopsis homologs.

TABLE S2 The transcriptional profiling of Fds and FdC1 on microarray data and RNA-seq analysis.

TABLE S3 List of oligonucleotides employed in this study.

## Abbreviations

Fd: ferredoxin
FdC: ferredoxin C
FNR: ferredoxin-NADP^+^ oxidoreductase
FTR: ferredoxin-thioredoxin reductase
NdhS: NADH dehydrogenase-like complex S
NiR: nitrite reductase
PGRL1: PGR5-like protein
PSI: photosystem I
SiR: sulfite reductase
