# Supplementary figures and images for: FdC1 and Leaf-Type Ferredoxins Channel Electrons From Photosystem I to Different Downstream Electron Acceptors
Source: Front Plant Sci. 2018 Apr 4;9:410. doi: 10.3389/fpls.2018.00410 (PMC5893904; doi:10.3389/fpls.2018.00410)

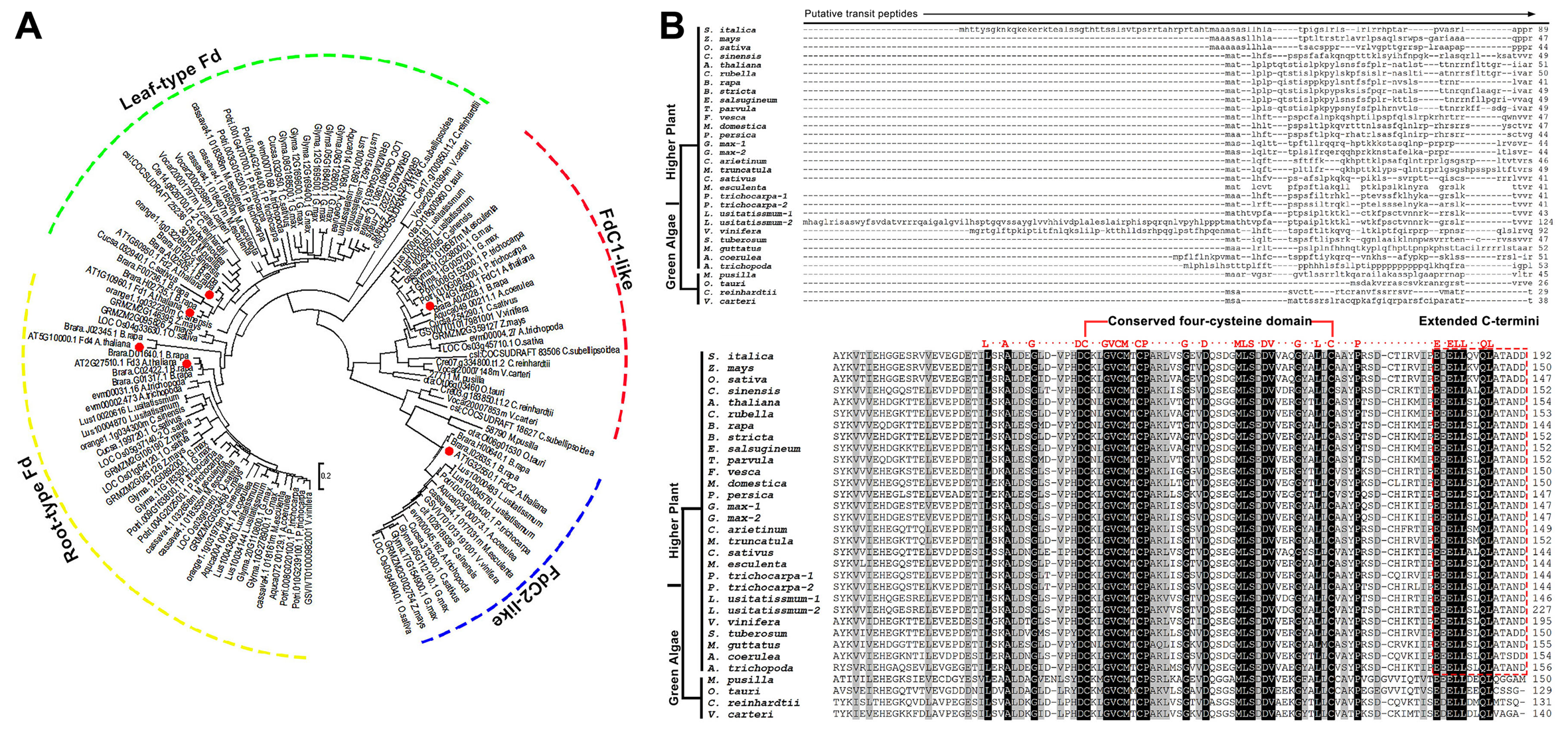

Supplement: FIGURE S1 — (A) Phylogenetic analysis of FdCs. The evolutionary history was inferred using the Neighbor-Joining method of MEGA7. The tree was drawn to scale, with branch lengths in the same units as those of the evolutionary distances. The evolutionary distances were computed using the Poisson correction method and are in the units of the number of amino acid substitution per site. 202 amino acid sequences of the putative mature proteins of the four isoforms of AtFd (Fd1∼Fd4) and two isoforms of AtFdC (FdC1 and FdC2) from 33 species were analyzed. All positions containing gaps and missing data were eliminated. There were in total 86 sequences in the final dataset. The ferredoxin and ferredoxin-like homologs could be divided into four clusters: leaf-type Fd (green dashed line), root-type Fd (dark red dashed line), FdC1-type (orange dashed line), and FdC2-type (blue dashed line). The Fd and FdC isoforms in A. thaliana are marked with red dots. (B) the multiple alignment of FdC1 from A. thaliana and its homologs from the other 28 species. A. trichopoda, AmTr_v1.0_scaffold00004.27; A. thaliana, AT4G14890.1; A. coerulea, Aquca_049_00211.1; B. stricta, Bostr.0597s0118.1; B. rapa, Brara.A02028.1; C. rubella, Carubv10007616m; C. reinhardtii, Cre07.g334800.t1.2; C. arietinum, Ca_11871; C. sinensis, cit_102630095; C. sativus, Cucsa.254290.1, E. salsugineum, Thhalv10026483m; F. vesca, mrna09776.1; G. max, Glyma.01G238000.1, Glyma.11G005700.1, L. usitatissmum, Lus10006116, Lus10010557; M. domestica, MDP0000155343; M. esculenta, cassava4.1_018587m; M. truncatula, Medtr5g006320.1; M. pusilla, 27711; M. guttatus, Migut.N00186.1; O. sativa, LOC_Os03g45710.1; O. tauri, Ot06g03460; P. trichocarpa, Potri.010G087300.1, Potri.008G153200.1; P. persica, ppa012963m; S. italica, Si037632m; S. tuberosum, PGSC0003DMT400086140; T. parvula, Tp7g13030; V. vinifera, GSVIVT01017981001; V. carteri, Vocar20007148m; Z. mays, GRMZM2G359127_T01. Identical residues are colored in white and highlighted in black back [file Image_1.TIF]

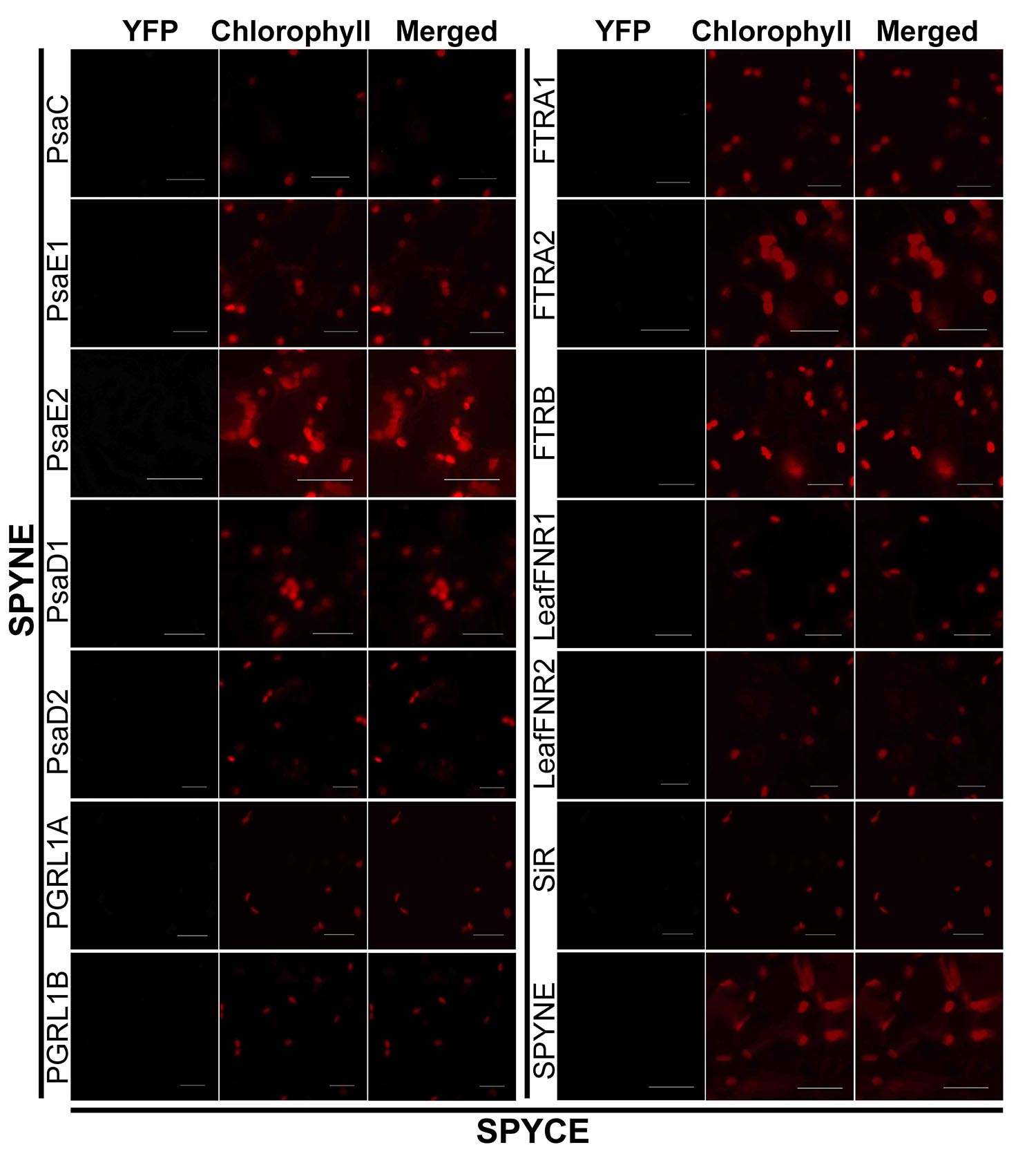

Supplement: FIGURE S2 — Negative control of BiFC. The assay was carried out by co-transforming agrobacteria with empty pSPYCE-35S and pSPYNE-35S vectors containing the coding sequences of the candidate proteins into tobacco leaves. The same laser scanning settings of Figure 3B were used to monitor the reconstituted YFP signals and chlorophyll autofluorescence. No YFP signal was detected (Scale bars: 20 μm). [file Image_2.JPEG]

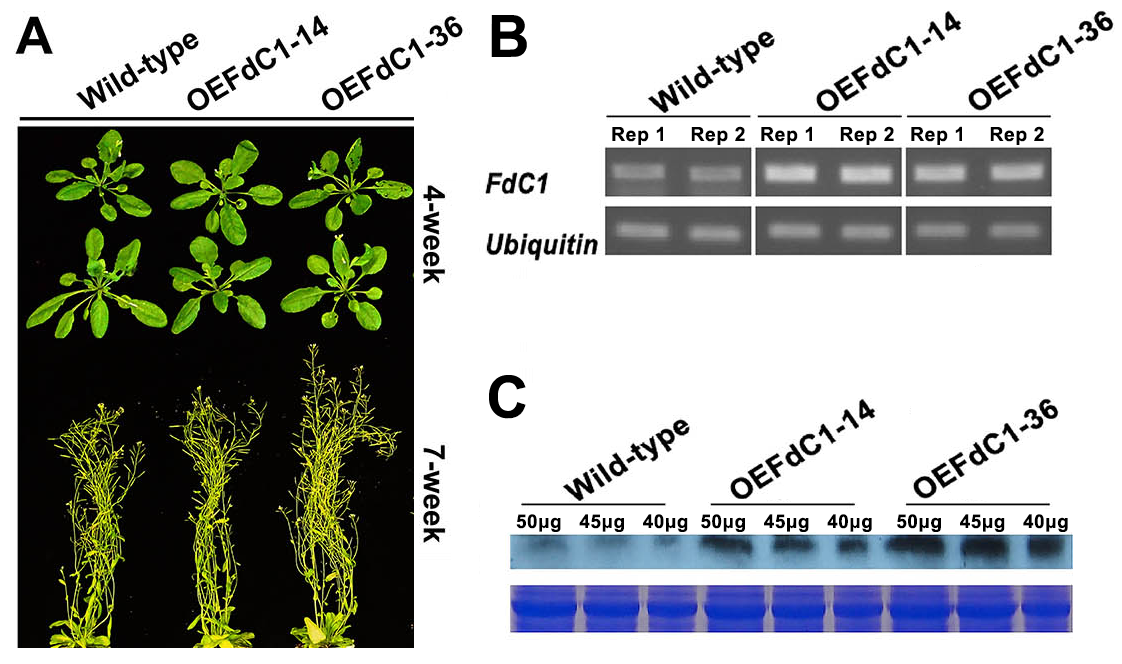

Supplement: FIGURE S3 — Growth phenotypes of FdC1 overexpression lines. (A) The phenotypes of the overexpression (OE) lines of FdC1 (OEFdC1-14 and OEFdC1-36) compared with the wild-type (WT). The vertical views of representative 4-week-old Arabidopsis and side views of representative 7-week-old plants are shown. All the plants were grown under long-day light regimes. (B) The semi-quantitative RT-PCR results showing the overexpression of FdC1 in transgenic lines at transcripts level. The primers were listed in Supplementary Table S3. The house-keeping gene ubiquitin 11 was chosen as the control. (C) The western blotting results showing the overexpression of FdC1 in transgenic lines compared with the wild-type. The Coomassie blue stain of Rubisco in SDS-PAGE gels was used as the control for equal loading. [file Image_3.TIF]
