# Supplementary material for: FdC1 and Leaf-Type Ferredoxins Channel Electrons From Photosystem I to Different Downstream Electron Acceptors
Source: Front Plant Sci. 2018 Apr 4;9:410. doi: 10.3389/fpls.2018.00410 (PMC5893904; doi:10.3389/fpls.2018.00410)
Supplement: TABLE S1 — Interacting amino acid residues of the two interacting proteins in resolved crystal structures and their corresponding residues in Arabidopsis homologs. [file Table_1.DOCX]

Table S1. Interacting amino acid residues of the two interacting proteins in resolved crystal structures and their corresponding residues in Arabidopsis homologs. The residues involved in the interactions between Fd and their corresponding partners (FNR, NiR, SiR, and FTR) were obtained from the well-resolved structures in the left two columns. The corresponding residues in their Arabidopsis homologs determined by multiple sequence alignment are listed in the right four columns. The sites of these residues are numbered from the N-termini of the putative mature proteins, and the sites in full-length amino acid sequences are shown in parentheses. Highly-conserved amino acids are shown in bold. Blank residues are shown as “-”.

| **1GAQ** | | **AtLFNR2** | **AtFd2** | **AtFdC1** |
| --- | --- | --- | --- | --- |
| **FNR** | **Fd** |  |  |  |
| K304 | E29 | **K306(359)** | **E29(81)** | L30(79) |
| E154 | R40 | **E156(209)** | **R40(92)** | N41(90) |
| K33 | D60 | R35(88) | **D60(112)** | G62(111) |
| K91 | D65 | **K93(146)** | **D65(117)** | S65(114) |
| K88 | D66 | **K90(143)** | **D66(118)** | **D66(115)** |
| K85 | A98 | **K87(140)** | - | L98(147) |

| **2AKJ-1A70** | | **AtNiR** | **AtFd2** | **AtFdC1** |
| --- | --- | --- | --- | --- |
| **NiR** | **Fd** |  |  |  |
| K49 | F63  E93  E94 | **K47(72)** | **F63(115)**  **E92(144)**  **E93(145)** | M63(112)  **E92(141)**  **E93(142)** |
| K80 |  | **K78(103)** |  |  |
| K83 |  | **K81(106)** |  |  |
| K100 |  | **K98(123)** |  |  |
| K266 |  | **K266(291)** |  |  |
| N304 |  | **N302(327)** |  |  |
| R502 |  | **R500(525)** |  |  |

| **5H92** | | **AtSiR** | **AtFd2** | **AtFdC1** |
| --- | --- | --- | --- | --- |
| **SiR** | **Fd** |  |  |  |
| R111 | D60 | **R49(121)** | **D60(112)** | G62(111) |
| R114 |  | **R52(124)** |  |  |
| R324 | D34 | **R261(333)** | **D34(86)** | **D35(84)** |
| K582 | E29 | **K519(591)** | **E29(81)** | L30(79) |
| K584 | E30 | **K521(593)** | **E30(82)** | D31(80) |

| **2PVG** | | **AtFTRB** | **AtFd2** | **AtFdC1** |
| --- | --- | --- | --- | --- |
| **FTRB** | **Fd** |  |  |  |
| K47 | E92 | **K39(77)** | **E92(144)** | **E92(141)** |
| S52 | Y37 | A44(82) | **Y37(89)** | **Y38(87)** |
| N73 | S38 | **N65(103)** | **S38(90)** | D39(88) |
| Q20 | Q68 | **Q12(50)** | **Q68(120)** | V68(117) |
| R24 | L64 | **R16(54)** | **L64(116)** | **L64(113)** |
| P75 | S62 | **P67(105)** | **S62(114)** | G62(111) |
