# Supplementary material for: FdC1 and Leaf-Type Ferredoxins Channel Electrons From Photosystem I to Different Downstream Electron Acceptors
Source: Front Plant Sci. 2018 Apr 4;9:410. doi: 10.3389/fpls.2018.00410 (PMC5893904; doi:10.3389/fpls.2018.00410)
Supplement: TABLE S2 — The transcriptional profiling of Fds and FdC1 on microarray data and RNA-seq analysis. [file Table_2.DOCX]

Table S2. The transcriptional profiling of Fds and FdC1 on microarray data and RNA-seq analysis.

The transcriptional microarray data in shoots and roots of 20-day-old wild-type Arabidopsis was retrieved from the supplementary files of Sun et al. (1), and the transcriptome data of the total RNA extracted from leaves of 20-day-old wild-type Arabidopsis at different time points based on RNA-seq technique was retrieved from the supplementary files of Liang et al. (2). The different time points are shown as T_0_ (end of night), T_1_ (one hour after onset of illumination), and T_8_ (eight hours after onset of illumination).

| **Name** | **Locus** | **Microarray signal** | | **RNA-seq (RPKM)** | | | |
| --- | --- | --- | --- | --- | --- | --- | --- |
|  |  | **Shoot** | **Root** | **Gene length** | **T_0_** | **T_1_** | **T_8_** |
| **AtFd1** | *AT1G10960* | 18722 | 592 | 654 | 569 | 1138 | 407 |
| **AtFd2** | *AT1G60950* | 55170 | 9400 | 833 | 2632 | 3242 | 3210 |
| **AtFd3** | *AT2G27510* | 1413 | 8367 | 754 | 5 | 4 | 3 |
| **AtFd4** | *AT5G10000* | 519 | 252 | 447 | - | - | - |
| **AtFdC1** | *AT4G14890* | 29090 | 3265 | 625 | 70 | 79 | 96 |

**REFERENCE**

1. Sun, F., Liang, C., Whelan, J., Yang, J., Zhang, P., and Lim, B. L. (2013) Global transcriptome analysis of AtPAP2 - overexpressing Arabidopsisthaliana with elevated ATP. *BMC Genomics* **14**, 752-752

2. Liang, C., Cheng, S., Zhang, Y., Sun, Y., Fernie, A. R., Kang, K., Panagiotou, G., Lo, C., and Lim, B. L. (2016) Transcriptomic, proteomic and metabolic changes in Arabidopsis thaliana leaves after the onset of illumination. *BMC plant biology* **16**, 1
