# Supplementary material for: FdC1 and Leaf-Type Ferredoxins Channel Electrons From Photosystem I to Different Downstream Electron Acceptors
Source: Front Plant Sci. 2018 Apr 4;9:410. doi: 10.3389/fpls.2018.00410 (PMC5893904; doi:10.3389/fpls.2018.00410)
Supplement: TABLE S3 — List of oligonucleotides employed in this study. [file Table_3.DOCX]

**Table S3. List of oligonucleotides employed in this study**

| **Vector**  **(Purpose)** | **Primer Name**  **(AGI code)** | **Primer Sequence (5’-3’)^1^** |
| --- | --- | --- |
| pGADT7  (Yeast two-hybrid) | PsaC  (ATCG01060) | F: ATTACATATGATGTCACATTCAGTAAAAATT  R: ATTACTCGAGTCAATAAGCTAGACCCATAC |
| pGADT7  (Yeast two-hybrid) | PsaD1  (AT4G02770) | F: ATTACATATGATGGCAACTCAAGCCGCCGG  R: GCTACTCGAGTTACAAATCATAACTTTGTTTGCCAG |
| pGADT7  (Yeast two-hybrid) | PsaD2  (AT1G03130) | F: ATTACATATGATGGCAACTCAAGCCGCCGG  R: GTCTCGAGTTACAAATCATAAGATTGTTTCCCAGTG |
| pGADT7  (Yeast two-hybrid) | PsaE1  (AT4G28750) | F: ATTACATATGATGGCGATGACGACAGCATC  R: CTTACTCGAGTTAAGCTGCAACTTCTTCGACCT |
| pGADT7  (Yeast two-hybrid) | PsaE2  (AT2G20260) | F: ATTACATATGATGGCGATGACGTCAGCAGC  R: GTTACTCGAGTCATTTTACTTCTTCCACCTCGTCC |
| pGADT7  (Yeast two-hybrid) | LeafFNR1  (AT5G66190) | F: TTAGCATATGATGGCTGCTGCTATAAGTGC  R: TACGCTCGAGTTAGTAGACTTCAACATTCCACTG |
| pGADT7  (Yeast two-hybrid) | LeafFNR2  (AT1G20020) | F: ATTACATATGATGGCGACTACCATGAATGC  R: TCTACTCGAGTCAGTAGACTTCAACGTTCCATT |
| pGADT7  (Yeast two-hybrid) | RootFNR1  (AT3G05390) | F: ATTGCATATGATGGCTCTCTCAACTACTCC  R: GCTACTCGAGTCAATACACTTCAACATGCC |
| pGADT7  (Yeast two-hybrid) | RootFNR2  (AT1G30510) | F: ATTGCATATGATGTCTCACTCTGCTGTTTCT  R: CTTACTCGAGTCAATAGACTTCAACGTGCC |
| pGADT7  (Yeast two-hybrid) | LeafFd1  (AT1G10960) | F: ATTACATATGATGGCTTCCACTGCTCTCTCC  R: ATTACTCGAGTTACATAATGGCTTCTTCTTTGTGG |
| pGADT7  (Yeast two-hybrid) | LeafFd2  (AT1G60950) | F: ATTGCATATGATGGCTTCCACTGCTCTCTC  R: GTCACTCGAGTTAAACAATGTCTTCTTCTTTGTGG |
| pGADT7  (Yeast two-hybrid) | PGR5  (AT2G05620) | F: TAATCATATGGCTGCTGCTTCGATTTCT  R: TAATCTCGAGCTAAGCAAGGAAACCAAGCCT |
| pGADT7  (Yeast two-hybrid) | PGR5-Like1A  (AT4G22890) | F: ATTGCATATGATGGGTAGCAAGATGTTGTT  R: TAATCTCGAGTTAAGCTTGGCTTCCTTCTG |
| pGADT7  (Yeast two-hybrid) | PGR5-Like1B  (AT4G11960) | F: ATTGCATATGATGGCTTTTACTCTAACAATCC  R: AACTGAGCTCTTAAGCTTTCCCTCCTTCTG |
| pGADT7  (Yeast two-hybrid) | FTRA1  (AT5G23440) | F: ATTGCATATGATGAGTAGCCAAATCGCTTTGT  R: TACTGAGCTCTCACTGATCAATGAACTCGAACTC |
| pGADT7  (Yeast two-hybrid) | FTRA2  (AT5G08410) | F: ATTGCATATGATGACTAACAGTTACGCTCTGTC  R: ATTACTCGAGTCACGGATCAATTAACTCGAAC |
| pGADT7  (Yeast two-hybrid) | FTRB  (AT2G04700) | F: AGCTGAATTCATGAATCTTCAAGCTGTTTC  R: ATCGCTCGAGTCACATGTTAGCTGTAGTTTC |
| pGADT7  (Yeast two-hybrid) | NdhS  (AT4G23890) | F: CCGGCATATGGCGACTTCTTCGATCACTAT  R: TAATCCCGGGTTATGGTGCTGCCTCTTCCT |
| pGADT7  (Yeast two-hybrid) | NiR  (AT2G15620) | F: ATTACATATGACTTCTTTCTCTCTCACT  R: ATTACCCGGGTCAATCTTCATTCTCTTCTC |
| pGADT7  (Yeast two-hybrid) | SiR  (AT5G04590) | F: ATTACATATGATGTCATCGACGTTTCGAGC  R: ATTACTCGAGTCATTGAGAAACTCCTTTGTATGT |
| pGBKT7  (Yeast two-hybrid) | FdC1  (AT4G14890) | F: ATTACATATGATGGCGACTCTTCCTCTCCCG  R: ATTACTGCAGTTAGTCGTTGGCAGTGGCGAG |
| pGBKT7  (Yeast two-hybrid) | FdC1 (1-145a.a.)  (AT4G14890) | F: ATTACATATGATGGCGACTCTTCCTCTCCCG  R: TAATCTGCAGTTACAAGAGTTCTTCCTCAGGGAT |
| SPYNE  (BiFC) | PsaC  (ATCG01060) | F: ATGCTCTAGAATGTCACATTCAGTAAAAATTT  R: ATTACTCGAGATAAGCTAGACCCATACTTCG |
| SPYNE  (BiFC) | PsaD1  (AT4G02770) | F: ATTATCTAGAATGGCAACTCAAGCCGCCGG  R: TAATCTCGAGCAAATCATAACTTTGTTTGCCAGT |
| SPYNE  (BiFC) | PsaD2  (AT1G03130) | F: ATTATCTAGAATGGCAACTCAAGCCGCCGG  R: TAATCTCGAGCAAATCATAAGATTGTTTCCCAGT |
| SPYNE  (BiFC) | PsaE1  (AT4G28750) | F: GAACTCTAGAATGGCGATGACGACAGCAT  R: ATTACTCGAGAGCTGCAACTTCTTCGACCTC |
| SPYNE  (BiFC) | PsaE2  (AT2G20260) | F: ATTATCTAGAATGGCGATGACGTCAGCAGC  R: TAATCTCGAGTTTTACTTCTTCCACCTCGTCCAAT |
| SPYNE  (BiFC) | FTRA1  (AT5G23440) | F: ATTATCTAGAATGAGTAGCCAAATCGCTTT  R: ATTACTCGAGCTGATCAATGAACTCGAACTC |
| SPYNE  (BiFC) | FTRA2  (AT5G08410) | F: CTAGTCTAGAATGACTAACAGTTACGCTCTGTC  R: ATTACTCGAGCGGATCAATTAACTCGAACTC |
| SPYNE  (BiFC) | FTRB  (AT2G04700) | F: ATTATCTAGAATGAATCTTCAAGCTGTTTCTTG  R: ATTACTCGAGCATGTTAGCTGTAGTTTCTTTTATT |
| SPYC/NE  (BiFC) | LeafFd1  (AT1G10960) | F: ATTATCTAGAATGGCTTCCACTGCTCTCTCC  R: TAATCTCGAGCATAATGGCTTCTTCTTTGTGG |
| SPYC/NE  (BiFC) | LeafFd2  (AT1G60950) | F: ATTGTCTAGAATGGCTTCCACTGCTCTCTC  R: ATTACTCGAGAACAATGTCTTCTTCTTTGTGGG |
| SPYNE  (BiFC) | LeafFNR1  (AT5G66190) | F: ATTATCTAGAATGGCTGCTGCTATAAGTGCT  R: TAATCTCGAGGTAGACTTCAACATTCCACTGTT |
| SPYNE  (BiFC) | LeafFNR2  (AT1G20020) | F: ATTATCTAGAATGGCGACTACCATGAATGC  R: TAATCTCGAGGTAGACTTCAACGTTCCATTGC |
| SPYNE  (BiFC) | PGR5-Like1A  (AT4G22890) | F: ATTATCTAGAATGGGTAGCAAGATGTTGTTT  R: TAATCTCGAGAGCTTGGCTTCCTTCTGGC |
| SPYNE  (BiFC) | PGR5-Like1B  (AT4G11960) | F: ATTATCTAGAATGGCTTTTACTCTAACAATCC  R: TAATACTAGTAGCTTTCCCTCCTTCTGGTAAT |
| SPYNE  (BiFC) | NiR  (AT2G15620) | F: TAATACTAGTATGACTTCTTTCTCTCTCACT  R: TAATCCCGGGATCTTCATTCTCTTCTCTTT |
| SPYNE  (BiFC) | SiR  (AT5G04590) | F: ATTATCTAGAATGTCATCGACGTTTCGAGC  R: TAATCTCGAGTTGAGAAACTCCTTTGTATGTATC |
| SPYC/NE  (BiFC) | FdC1  (AT4G14890) | F: ATTATCTAGAATGGCGACTCTTCCTCTCCCG  R: ATTACTCGAGGTCGTTGGCAGTGGCGAGCT |
| pRS300  (RNAi line Overlapping PCR) |  | A: CTGCAAGGCGATTAAGTTGGGTAAC  B: GCGGATAACAATTTCACACAGGAAACAG |
| pCXSN  (TA clone OE line) | FdC1  (AT4G14890) | F: ATGGCGACTCTTCCTCTCCCG  R: TTAGTCGTTGGCAGTGGCGAG |
| pBA002-eYFP  (Subcellular location) | FdC1  (AT4G14890) | F: TAATCTCGAGATGGCGACTCTTCCTCTCCC  R: TAATCTCGAGGTCGTTGGCAGTGGCGAGCT |
| pGEX-6P-1  (Express protein) | LeafFd2  (AT1G60950) | F: TAATGGATCCATGGCTACATACAAGGTCAAG  R: TAATCTGCAGTTAAACAATGTCTTCTTCTTTGT |
| pGEX-6P-1  (Express protein) | FdC1  (AT4G14890) | F: TAATGGATCCATGGCTCGGGCATATAAAGT  R: TAATCTGCAGTTAGTCGTTGGCAGTGGCG |
| pGEX-6P-1  (Express protein) | OAS-TLC  (AT3G59760) | F: TAATGGATCCATGGCTGTTAAGCGCGAGACTG  R: TAATGTCGACCTAATGTTCATACCTCAGGCTGCAT |
| semi quantitative RT-PCR | LeafFd2  (AT1G60950) | F: CTTCATTCATCCGTCGTTCC  R: AGGGTAAGCAGCACAAGTGA |
| semi quantitative RT-PCR | FdC1  (AT4G14890) | F: CCGTAACGCAACACTCTCAA  R: ACGAGTTTCGCTGGACAAGT |
| semi quantitative RT-PCR | Ubiquitin 11  (AT4G05050) | F: GCAGATTTTCGTTAAAACC  R: CCAAAGTTCTGCCGTCC |

*The restricted digestion sites were underlined
